# Helicopter EMS: Research Endpoints and Potential Benefits

**DOI:** 10.1155/2012/698562

**Published:** 2011-12-01

**Authors:** Stephen H. Thomas, Annette O. Arthur

**Affiliations:** Department of Emergency Medicine, University of Oklahoma School of Community Medicine, OU Schusterman Center, 4502 East 41st Street Suite 2E14, Tulsa, OK 74135-2553, USA

## Abstract

Patients, EMS systems, and healthcare regions benefit from Helicopter EMS (HEMS) utilization. This article discusses these benefits in terms of specific endpoints utilized in research projects. The endpoint of interest, be it primary, secondary, or surrogate, is important to understand in the deployment of HEMS resources or in planning further HEMS outcomes research. The most important outcomes are those which show potential benefits to the patients, such as functional survival, pain relief, and earlier ALS care. Case reports are also important “outcomes” publications. The benefits of HEMS in the rural setting is the ability to provide timely access to Level I or Level II trauma centers and in nontrauma, interfacility transport of cardiac, stroke, and even sepsis patients. Many HEMS crews have pharmacologic and procedural capabilities that bring a different level of care to a trauma scene or small referring hospital, especially in the rural setting. Regional healthcare and EMS system's benefit from HEMS by their capability to extend the advanced level of care throughout a region, provide a “backup” for areas with limited ALS coverage, minimize transport times, make available direct transport to specialized centers, and offer flexibility of transport in overloaded hospital systems.

## 1. Introduction

This discussion strives to overview potential benefits accrued by utilization of helicopter EMS (HEMS). The goal will be to outline the major HEMS-associated gains accrued by patients, EMS systems, and healthcare regions.

The existence and degree of benefits from HEMS use have been debated for years. Unfortunately, it is not possible to know, at the time of vehicle triage, precisely which patients will benefit from HEMS. On the other hand, there are systematic reviews of the literature which strongly suggest that HEMS accrues benefits for at least some types of patients [1–3]. An example review is found in a 2007 report from the independent Institute of Health Economics, prepared for the Canadian health ministry in Alberta. These authors, after reviewing all available studies from the year 2000, concluded: “Overall, patients transported by helicopter showed a benefit in terms of survival, time interval to reach the healthcare facility, time interval to definite treatment, better results, or a benefit in general.” [4].

Since few would argue that HEMS benefit is always predicated solely on time and logistics, any consideration of HEMS outcomes must include broader considerations of out-of-hospital care (for purposes of consistency within this paper, “prehospital” is interchangeable with “out-of-hospital” in order to encompass both scene and interfacility transports). The HEMS crews' extended practice scope offers circumstances well suited for assessing high-level advanced life support (ALS) care and its potential benefits [5]. For example, studies assessing prehospital intubation (ETI) have provided important—if unintended—insight into HEMS' salutary impact on outcome [6–8]. 

Many questions remain unanswered about HEMS. However, there is a growing body of evidence addressing HEMS' potential benefits. This paper aims to assist those working toward proper deployment of air medical resources. The discussion also aims to aid those planning further HEMS outcomes research. 

## 2. Outcomes Assessment in HEMS

Various outcomes will be visited in detail later in this paper, but it is appropriate to commence with a brief word on what constitutes an “outcome.” This paper will address primary, secondary, and surrogate outcomes. 

### 2.1. Primary Outcome Variables: Survival and Functional Outcome

The most important outcome for HEMS studies is that of functional survival. This is the primary outcome addressed in most studies referenced in this paper. Since survival to hospital discharge in a persistent vegetative state is different from functional survival, consideration of neurological condition should be (and usually is) incorporated into survival definition.

Discussions of HEMS' potential utility often mention safety. The line of thinking, usually advanced by those who believe that HEMS is significantly overused, is that against any potential benefit accrued by HEMS should be weighed the risks associated with air transport. The importance of safety as the prime consideration in HEMS is undeniable, but the subject is beyond the scope of this paper. Readers are directed to other expert resources, who have assessed prehospital air and ground vehicle safety [9, 10]. 

### 2.2. Secondary and Surrogate Outcome Variables

These variables either explore possible mechanisms for morbidity/mortality reduction (e.g., peri-intubation physiology), or assess parameters that are indirectly linked to outcomes improvement (e.g., time to cardiac catheterization).

One outcome of increasingly recognized importance is relief of pain. Though listed here as a secondary variable, pain relief has been considered by many EMS experts as a stand-alone (i.e., primary) outcome for prehospital care [11]. 

An additional set of secondary variables encountered in the medical literature deals with lengths of stay in various hospital departments (e.g., ICU stay). The problems attendant to use of these endpoints are well known to clinical researchers; length of stay is impacted by many factors well downstream from transport modality. Nonetheless, at least one HEMS study [12] suggests that, as compared with ground-transported cardiac patients, those transported by air had a 2-day decrease in hospital length of stay due to improved myocardial salvage. That study is the exception to a general rule that HEMS literature focuses more on mortality and other surrogate endpoints (see below) rather than addressing hospital lengths of stay. 

Studies assessing HEMS' impact on surrogate endpoints constitute an important set of contributions to the literature. Surrogate variables tend to be physiologic (e.g., hypoxemia, hypercapnia, hypotension) or logistic (e.g., prehospital time, time to advanced care) parameters with evidence basis for delineation as endpoints. Some surrogate outcomes are not likely to be testable in terms of precise mortality benefit; an example would be the ability of highly trained HEMS crews to streamline interfacility transports of ruptured abdominal aortic aneurysm patients by taking them directly to receiving hospitals' operating rooms [13]. Some secondary and surrogate endpoints may lack universal acceptance as impacting functional outcome, but others (e.g., hypoxemia in brain-injured patients) are solidly evidence based [14]. 

An emerging view is that patient safety initiatives in HEMS have resulted in low incidence of adverse events during air medical transport. Given the importance of patient safety, and the increasing attention to this as an “outcome,” preliminary work such as that by MacDonald et al. [15] is of vital import. Assessment of patient safety and adverse events is in early stages, but HEMS investigators deserve credit for focusing on this measure.

### 2.3. Nontrauma Outcomes Analysis

Nontrauma outcomes studies are limited largely for two reasons: (1) experts in many countries, even where there is debate over HEMS' effectiveness, are in agreement that a randomized controlled trial of HEMS versus ground transport is not currently feasible, and (2) since HEMS-triaged patients tend to be of higher acuity than ground-transported patients, outcomes analysis has to be acuity adjusted; however there is no consistently reliable means of adjusting for acuity in HEMS-transported nontrauma patients.

Another issue with nontrauma outcomes studies is that the transport population comprises disparate diagnostic groups (e.g., pregnancy, acute coronary syndromes, epiglottitis, stroke, poisoning). Such disparity translates into small numbers for a single diagnosis, leading to biostatistical difficulties in detecting a small but clinically important benefit in the less commonly encountered patient types.

As a result of the issues noted above, use of HEMS for nontrauma patients has an evidence base that is, compared to that for trauma transport, less concrete. Nonetheless, there is evidence of various sorts. Absent controlled trials, some consideration should be given to expert opinion and even (for unusual diagnoses) case reports. These reports are often based upon time savings accrued with HEMS. Some examples follow, for cardiac, stroke, and “sporadic-use” cases.

An editorial [16] appearing in Chest, the journal of the American College of Chest Physicians, observed that “in many communities, emergency air medical systems have become an integral part of the practice of cardiology and critical care medicine.” The authors go on to aver that “we firmly believe that air medical transport is a safe means for transport of cardiac patients and should be considered for patients who require transfer to more specialized centers for additional diagnostic and therapeutic interventions.” Reports outlining extension of percutaneous coronary intervention to community hospitals include incorporation of HEMS into systems planning, as a necessary backup in cases where urgent CABG is required [17]. It is increasingly well known that time savings—in the manner that may be achieved by judicious HEMS use—can be helpful: each 30 minutes' additional ischemia time increases mortality by 8–10%. [18] Additionally, work from the TRANSFER-AMI group suggests that expedited transfer for mechanical intervention after community hospital lysis is associated with a 50% reduction in the 30-day composite endpoint (death, reinfarction, recurrent ischemia/reinfarction, CHF, or shock) [19]. It seems likely that HEMS will occasionally be a valuable option for some patients receiving this combined-therapy approach.

Similar to the situation with integration of HEMS into cardiac care systems is the rapidly solidifying role for air transport in stroke care. A Resource Document for a position statement of the National Association of EMS Physicians recommends air transport of stroke patients if the closest fibrinolytic-capable facility is more than an hour away by ground [20]. The American Stroke Association Task Force on Development of Stroke Systems [21] identified HEMS as an important part of stroke systems. The report states “air transport should be considered to shorten the time to treatment, if appropriate.” Authors writing about the utility of HEMS in stroke (and also cardiac) care systems generally refer to the ability, addressed in detail later in this discussion and bolstered by logistics studies, of HEMS to “extend the reach” of tertiary care centers providing time critical care [22, 23]. A potential role for time critical transport in improving stroke outcomes is suggested by the pooled analysis revealing a stepwise outcomes improvement associated with each 90-minute improvement in lysis time (to 270 minutes) [24]. 

Another type of difficult-to-categorize (and equally hard to research) “outcomes” publication is the case report. As an example of many such reports, there is a description [25] of lifesaving HEMS use in a 32-year-old ARDS patient who received inhaled prostacyclin during an air medical transport that was deemed to be critical to that patient's survival. Others, in both the U.S. and abroad, have highlighted the occasional utilization of HEMS (scene runs) to enable stroke patients to reach specialized care centers in time to receive outcome-improving lytic therapy [26, 27]. Recently, a Canadian group described use of air transport to get critically needed antidotes (fomepizole in one patient, Digibind in another) to patients up to 6 hours faster than would have been the case had therapy been delayed to ultimate arrival at receiving centers [28]. 

### 2.4. Trauma Outcomes Analysis

Trauma outcomes analysis has a major advantage over nontrauma outcomes analysis in that there are more transported patients with trauma; this allows for more robust statistical methods. Also, there are many scoring systems (e.g., Glasgow Coma Score [GCS], Trauma Score [TS], Injury Severity Score [ISS]) that can be used to stratify patient acuity.

The capability of scoring systems to adjust, at least partially, for differences in patient acuity translates into an improved ability to combine patients from many HEMS programs and thus conduct multicenter research. Since most (though not all) HEMS programs transport a majority of trauma patients, the larger numbers for injured patients mean that it is easier to conduct outcomes research on trauma patients than on other populations.

The reader is expected to be familiar with most of the simple scoring systems, such as GCS and TS, but one subject—TRISS—is more complicated. Since TRISS is frequently encountered in the HEMS literature, and since its application (and misapplication) has important implications for appropriate interpretation of many trauma outcomes studies, readers are referred to a more definitive source such as the work of Boyd et al. [29]. For the purposes of this paper, it is noteworthy that TRISS, while prone to misapplication (e.g., employment of nonstandardized analysis in a patient population with inappropriately low M statistic), remains the gold standard for predicting outcomes in trauma patients [30–32]. 

## 3. Potential HEMS Benefits to Patients

### 3.1. Mortality Improvement as an Endpoint

This seems like the most obvious potential benefit upon which to focus, and in fact survival improvement has been the main endpoint of most of the major HEMS studies. Mortality is a relatively concrete endpoint (the only vagaries being introduced by a postincident time demarcation, such as 30- or 60-day mortality). Mortality is also relatively easy to address in the large, retrospective study designs (usually registry based) that comprise much of the HEMS outcomes literature. [6, 33, 34] As a dichotomous, easily ascertained endpoint, mortality can also be assessed with novel techniques. One example is the artificial neural network methodology reported by Davis et al., who identified HEMS (as compared to ground transport) as saving a statistically significant 3.6 lives per 100 transports of brain-injured patients with head AIS of at least 3 (when analysis focused on patients with GCS 3–8, 7.1 lives were saved per 100 transports) [5].

There are some potential weaknesses to use of the mortality endpoint. For example, the time frame is often not clearly laid out in HEMS studies, and the mortality timeline can be defined arbitrarily (e.g., 30 or 60 days). Additionally, though mortality is undoubtedly the most important clinical endpoint, it is difficult to assess unless there are large patient numbers and some means of matching acuity in air- and ground-transported patients. Finally, most HEMS study designs performing isolated assessment of mortality do not isolate the mechanism (e.g., streamlined prehospital times, improved airway management) for HEMS benefit.

### 3.2. Morbidity Improvement as an Endpoint

Since mortality assessment provides an incomplete picture, it makes sense to ascertain whether any nonmortality endpoints are affected by transport mode. Even if HEMS does impact mortality, it likely does so at a sufficiently low frequency that detection is difficult (given methodological issues and need to control for acuity). It is possible that nonmortality endpoints are reached with greater frequency than survival improvement, and thus nonmortality endpoints may be easier to detect. Additionally, nonmortality endpoints may provide clues to the mechanism by which HEMS improves survival (e.g., decrease in aspiration pneumonia implying improved airway management).

There are weaknesses associated with assessing morbidity. Heterogeneity is one. Depending on disease process, a myriad of nonmortality endpoints could theoretically be assessed. The potential utility of the “strength in numbers” argument (i.e., that nonmortality endpoints occur more frequently than survival accrual) is somewhat offset by the fact that assessment of many nonmortality endpoints requires analysis of a subgroup of patients in a certain diagnostic category; such limitation of the focus of a study results in lower numbers of patients with the endpoint in question. (Sometimes there are still enough numbers for functional outcomes assessment. For head injuries there is clear evidence that HEMS improves these nonmortality outcomes [6, 35]). 

The literature includes some studies which address nonmortality endpoints such as quality-of-life and Glasgow neurological outcome score. It is fair to point out that those considering the weight of the evidence in favor of HEMS' improvement of trauma mortality have also stated the naturally following conclusion that HEMS improves morbidity, writing that appropriate air medical utilization will result in “lives saved and disabilities prevented” [36].

### 3.3. Secondary Endpoints

Secondary endpoints are myriad. The most useful are those with high clinical relevance. Examples are provided in this section.

Physiologic parameters are frequently used as secondary endpoints in HEMS studies. As mentioned earlier in this discussion, HEMS airway management for head injured patients has been shown to be associated with improved patient outcome [6, 37]. Investigators have addressed the intermediate mechanism for outcome improvement. It appears to be related to improved oxygenation and ventilatory practices, as reflected in peri-ETI (i.e., before and after ETI) physiologic parameters such as end-tidal CO_2_, which may be frequently disrupted during ground EMS ETI [38], whereas they are much less during HEMS ETI or even with HEMS transport postground ambulance ETI [39–42]. Evidence demonstrating deleterious impact of peri-ETI physiologic disruptions (in head injury patients, at least) is sufficiently compelling that studies, showing less such derangement in HEMS patients, should be considered as highly relevant to the outcomes debate. Recent analyses also demonstrate improved hemodynamic management, with investigators concluding that improved blood pressure management by HEMS was partially responsible for improved head injury outcome [35]. 

The next major secondary endpoint is pain control. After being neglected for too long as a priority for acute-care (and prehospital) medicine, the subject of pain care is finally receiving its due. Experts in prehospital care have written that pain care is a valid endpoint in and of itself [11, 43, 44]. Furthermore, the fact is that HEMS providers tend to be more diligent in assessing and treating pain, than ground EMS providers [11, 45]. 

While HEMS patients are different from ground EMS patients, the studies of patients with suspected isolated fractures result in substantial differences in analgesia rates (ranging from 1.8–12.5% for ground EMS, to over 90% for HEMS as outlined in detail in another review [43]). In fact, EMS experts writing about pain management have acknowledged the better HEMS performance with respect to analgesia provision, stating that (as compared with ground EMS) HEMS is characterized by a “population of patients and providers very different from ground EMS-transported patients” [46].

As HEMS researchers try and extend their outcomes assessments beyond mortality, pain assessment and care represent a fertile ground for (partial) justification for use of HEMS. In some patient populations, such as those with suspected myocardial infarction, pain control is a paramount clinical goal. Thus, assessment of potential benefits of HEMS should take into account studies finding better pain control in HEMS-transported cardiac patients—who are of higher acuity with commensurate increased likelihood of refractory pain—than those transported by ground [12]. It is easy to argue that good pain care can be brought to bear by ground EMS (i.e., analgesia is allowed for in protocols), but the existing evidence on what is done is consistent with a HEMS pain management benefit. 

### 3.4. Surrogate Endpoints

Distinction between endpoints that are secondary (as outlined above) and those that are surrogate (defined in this discussion as indirect mediators of improved outcome) can be tricky. The delineation is in some cases semantic. Examples of surrogate endpoints are provided in this section.

Earlier ALS care is one surrogate endpoint. Especially in rural or isolated areas, HEMS may represent the best means to get ALS to patients within a reasonable time frame. The significant improvement in “time to treatment” associated with HEMS utilization has been noted in systems throughout the world [47]. Though there is little data to actually prove that ALS improves outcomes, many EMS experts—and most systems benchmarkers—believe this to be an important goal for optimizing care of many types of patients. Furthermore, given the extant data showing that at least one ALS intervention—ETI—improves outcome when provided by HEMS, it naturally follows that the earlier provision of such an intervention will often be in the patient's best interests. More recent data, especially focusing on patients with severe trauma including head injuries, suggests that the earlier arrival of those capable of providing ALS-level airway and hemodynamic support translates into improved overall outcome and better neurological function [35]. Authors of case series demonstrating high rates of neurologically intact survival in diagnoses such as drowning also make strong arguments for the advantages of dispatching experienced ALS-level crews to areas in which such coverage (by ground EMS) is lacking [48]. Trauma specialists assessing nationwide data indicating HEMS outcomes improvement have written that although HEMS' logistics advantages may be uncommon in some areas, there are definitely regions within the US at least, where the access provided by HEMS is life saving [49]. 

A second surrogate endpoint is the extension of critical care experience and capabilities, throughout a region. In many regions, HEMS providers have pharmacological and procedural capabilities that outstrip the tools available to ground EMS. The differences in care capabilities can be dramatic. A report from the UK contended that patient outcomes were improved by performance of field thoracotomies [50]. Trauma surgeons reinforcing the concept of providing critical interventions during the “golden hour” have written that HEMS response to trauma scenes allows for provision of this life-saving care in timely fashion [51, 52]. Recent analysis of US data from the National Trauma Data Bank (NTDB) prompted authors to conclude that, on a nationwide level, one of the major advantages of HEMS is the higher level of care often provided by air medical crews [49].

In terms of air medical crew capabilities, the situation in the area served by Mayo Clinic in Minnesota may be reflective of what occurs in many areas. Ground EMS providers carry 20 different drugs but HEMS crews carry three times that number and can provide therapy such as blood transfusion and antibiotics for patients with open fractures [53]. Prehospital providers from some HEMS programs can provide advanced interventions such as tube thoracostomy; available evidence suggests that HEMS crew-placed chest tubes are as effective as, and no more likely to be associated with complications than, those placed in the hospital setting [54].

Especially in rural areas, the only prehospital care available may be BLS level [53]. It has been noted that since “HEMS brings a level of care to a trauma scene or small referring hospital that is over and above care rendered by an ALS ground ambulance,” many procedures such as intubation (even for patients transferred from referring hospitals) are deferred to the HEMS crew [53]. Thus, HEMS is an important mechanism for getting to the patient medical crews that bring important expertise to both trauma scenes and small community hospitals. For example, HEMS crews using neuromuscular blockade have long demonstrated ETI success rates that rival those achieved in the E.D., whereas outcomes with ground EMS ETI (even with neuromuscular blockade) tend to be worsened [6, 14, 37, 42, 53, 55–57]. It seems likely that poor results from ground ETI (as assisted by neuromuscular blockade) are related in part to provider inexperience and differences in training for HEMS as compared to most ground providers [6, 53, 58, 59]. As another example, critical care transport teams (that use both helicopter and ground vehicles) have reported sophisticated ventilatory and other advantages accrued with application and use of ETCO_2_ monitoring in pediatric and adult patients [40, 53, 60]. 

As mentioned previously, there is increasing evidence that better airway management skills are responsible for better outcomes in at least some patient groups. While this HEMS discussion is not the place for a detailed analysis of airway management issues, it is undoubtedly the case that failure to obtain or maintain a trauma patient's airway is a significant cause of preventable death [61].

For patients with head injury, there are now large-scale studies identifying markedly improved outcome for HEMS patients, as compared to ground ambulance transports, for those undergoing prehospital ETI [6, 37, 42]. Technically, ETI is better considered as an “ALS” level intervention, rather than an expanded practice scope maneuver. However, it is unrealistic to expect that the airway management expertise possessed by busy HEMS providers can be easily attained by practitioners with less experience and less rigorous training [58]. (What only the future can tell is whether HEMS crews will maintain their proficiency and high ETI success rates. There are few data, but anecdotal evidence suggests the possibility of skills dilution due to burgeoning numbers of air transport services and increasing difficulties in obtaining ETI experience in settings such as the operating room). 

The improved outcomes for head-injury patients transported by HEMS have also been suggested to be associated with other traditional ALS-level maneuvers that are simply performed better by highly trained air medical crews. In a discussion of possible explanations for HEMS-associated improved mortality and neurologic outcomes for HEMS (as compared to ground ALS), an Italian group [35] noted that not only were airways more commonly managed, but that IV access and fluid resuscitation were handled significantly better by HEMS. 

In addition to ETI capabilities, HEMS crews operate with benefit of experience dealing with critically ill and injured patients; the HEMS crews may in fact have more comfort with high-acuity patients than even physicians at a referring hospital [53, 61]. Analyses of HEMS systems have consistently revealed a relationship between crews' advanced training/experience and performance of critical tasks. In this era of focus on medical errors, it is noteworthy that recent studies (particularly in the pediatric population, but also in adults) have strongly suggested that errors (e.g., missed esophageal intubations, inadvertent extubation, incorrectly sized, or too-deeply-placed endotracheal tubes) are more likely in ground as compared to HEMS-transported patients [62]. There has been little concrete correlation between such findings and mortality, but the common-sense implications are difficult to refute. 

### 3.5. Streamlined Prehospital Times for Scene Missions

It is well known that, particularly for rural locations, prolonged EMS response/transport time results in increased trauma mortality [63]. Shorter transport duration would seemingly be a “given” for HEMS use, but ever since early HEMS studies (e.g., Baxt and Moody [64].) found that HEMS did not save time; this supposed HEMS benefit has been subject to debate. Studies conducted from regions as disparate as California and the Netherlands clearly demonstrate HEMS mortality benefit, yet find similar scene-to-trauma center times for ground and HEMS transports [65, 66]. One study from a well-developed HEMS and trauma system in the Netherlands finds that (physician-staffed) HEMS crews' scene times are about 10 minutes longer than those for ground EMS crews (25 versus 35 minutes in a system in which HEMS crews stabilize patients prior to ground transport to Level I care) [51]. The prolongation in scene times was accounted for by patient acuity and casemix, and adjusted analysis showed no effect of on-scene time on survival (OR 1.0, *P* = .89) [51]. In fact, the traumatologists assessing the slight prolongation in on-scene times associated with HEMS argue that bringing advanced interventions to the scene was actually a benefit to survival—the HEMS crews' interventions had sufficient salutary impact as to completely negative the well known adverse impact of prolonged prehospital times, on outcome [61]. 

The consideration of urban scene HEMS use is problematic. On the one hand, HEMS transport from areas close to a trauma center does not make much sense as a time saver since distances are short. On the other hand, such areas tend to be particularly prone to traffic congestion and transport delays (depending on the time of day). Since most studies focusing on transport times tend to have the times for one vehicle type or the other “estimated” it is easy for bias to be introduced. The fact that such transport times for either air or ground EMS are estimated retrospectively (i.e., lacking information about traffic or weather or other conditions) further dilutes the value of these estimates.

Faster transport time is, in some cases, potentially life saving—the premise that faster transport improves trauma mortality is widely accepted based upon available evidence. Authors focusing on logistics studies support the notion that time to definitive care is an appropriate primary endpoint, writing that “The correlation between length of time to definitive care and outcome has been well established in the literature, so the premise that faster transport is better seems justifiable[67].” The authors of a Pennsylvania trauma registry-based head injury study, in noting that HEMS was associated with improved survival and functional outcome, noted that their results could have “simply reflected the effect of [faster] transport time to trauma center” [6]. Furthermore, the impact of logistics on trauma mortality has been argued in a 2005 JAMA study which found that HEMS represented the only mechanism by which 27% of the US population had timely Level 1 or 2 trauma center access (within an hour of receipt of emergency call) [68]. Put another way, HEMS has been estimated to be the only mechanism by which 81.4 million Americans have timely (<1 hour) access to Level 1 or Level 2 trauma centers [68]. The authors concluded that new helipad placements and additional HEMS programs “could be an important, and practical, means of extending trauma center access to populations that currently have none.” Since the JAMA study group comprised both clinical and epidemiologic trauma systems leaders, their paper—with its assumption that HEMS is useful from a time-distance perspective—is a useful complement to the HEMS utility dialogue. The fact that HEMS provides the only timely access to high-level trauma care is particularly noteworthy, given recent large-scale studies finding that Level I trauma care results in a distinct outcomes benefit as compared to other levels of trauma care; [69, 70] for at least a quarter of the US population, helicopters thus represent the only mechanism for rapidly accessing life-saving care for injuries. 

Related conclusions about the critical utility of air medical transport for the US population have emanated from burn investigators. While there is no “golden hour” for burn patients, epidemiologists and clinicians writing in a JAMA study point out that early care (in the first few hours) at a burn center improves outcome and that HEMS is the sole mechanism by which millions of Americans have access to burn center care within 2 hours of injury [71]. 

### 3.6. Rapid Transport for Interfacility Missions

The idea of HEMS utilization to expedite care for patients with time critical injury and illness is not new. There is a significant body of literature addressed in this monograph and elsewhere, that demonstrates HEMS utility for time savings (and mortality advantage) in secondary (interfacility) trauma transport [72]. Recent nationwide database analysis reveals that HEMS use in the US is associated with significant time savings. Loss of HEMS availability has been recognized as a potentially important factor causing increased trauma mortality in patients presenting to non-Level I centers [73]. 

Besides use for trauma diagnoses, there is growing emphasis on employment of HEMS to expedite care for patients with time critical nontrauma illness. The utility of HEMS' logistics/speed capabilities to extend the reach of Level I centers' time-windowed advanced cardiac and stroke care has been the subject of increasing attention, with particular emphasis being given on the ability of HEMS to expedite care of these time-sensitive diagnoses [74, 75]. The use of air medical resources to rapidly move patients to specialized centers is gaining increasing attention in part because of the ever-growing realization that “time is myocardium”, “time is brain tissue”, and so forth [74, 76, 77]. 

In terms of cardiac patient transports and time savings, there is increasing emphasis on getting patients with myocardial infarction to primary PCI as the treatment of choice if a 90 minute first-door-to-balloon time can be met; expedited prehospital care—including HEMS—will play an important role in cardiac care systems [78, 79]. The 90-minute “window” is not absolute. Emergency Medicine experts have written that the maximal benefit of primary PCI is accrued in the initial 60 minutes [80]. In fact, it is known that each 15-minute decrement in time to PCI, from 150 minutes down to <90 minutes, is associated with 6.3 fewer deaths per 1000 patients treated [81]. Given these findings, it is hard to dispute the importance of data such as those from a rural setting in Pennsylvania. Investigators instituted a streamlined HEMS transport program for community hospitals to get patients into receiving center PCI, and tracked the proportions of patients with community hospital door to wire-crossing times under 90 minutes and 120 minutes. For both time frames, the proportions of patients meeting the timing endpoints increased significantly (under 90 minutes, from 0% to 24%; under 120 minutes, from 2% to 67%) [75]. Complementary to the results in Pennsylvania are data from Ohio, that demonstrate that having HEMS transport cardiac patients is no guarantee of arrival to cath labs within recommended time frames [82]. HEMS is potentially important as a part of a cardiac care system, but the air medical resource must be used wisely.

Cardiac patients are not the only nontrauma diagnosis for which time is critical. Considered in one easily understood way, each hour of ischemic stroke results in neuronal damage approximating 3.6 years of normal aging [77]. Furthermore, ED specialists have noted with concern the consistency of reports finding that nearly 1 in 5 patients receiving lysis for “stroke” based upon CT reading in fact have nonstroke “mimics” of acute thromboembolic CVA [83]. The increasing awareness that advanced imaging optimizes accuracy and safety, combined with the current (and likely near-future) lack of round-the-clock availability of such imaging [83], has high potential to translate into a major role for early and rapid HEMS transport for stroke. These time critical findings emphasize the importance of integrating HEMS into stroke care networks. Addition of air medical resources into logistics calculations halves the numbers of Americans who lack timely (within one hour) access to a primary stroke center (from 136 million to 63 million) [84].

Sepsis, a long-recognized disease process, has not generally been considered “time critical.” This view has changed, with the advent of studies demonstrating improved outcome associated with goal-directed therapy. Recent reviews of sepsis care have emphasized the importance of the six-hour goal for institution of high-level sepsis care [85]. Though many patients with sepsis do not undergo transport at all, HEMS may in some cases provide a useful mechanism for rapidly getting patients to appropriate, time critical, goal-directed therapy.

HEMS has also long been known to allow for maternal (and fetal) outcome benefits for high-risk obstetrics transports that simply would not have occurred (due to physician unwillingness to have prolonged transport times) in the absence of air transport [86]. More recently, a group from Florida [87] has reported that scene transports for suspected stroke patients resulted in extension of their stroke care to patients previously outside of the “logistics envelope”, and others have reported that HEMS is useful to expedite interfacility stroke and cardiac transports [88, 89]. 

One approach currently in investigative use in Boston incorporates the twin novelty of prehospital EKG triggering of both HEMS dispatch and activation of the receiving hospital's cardiac cath lab. The aircraft thus arrives at the (non-PCI-capable) community hospital within minutes of the patient's arrival by ground EMS [90]. After the community hospital's ED physician quickly confirms the diagnosis based upon review of the prehospital 12-lead EKG, the tertiary center's cardiac catheterization laboratory activation is confirmed, and the helicopter (either already at, or very close to, the community hospital) completes rapid transport directly to the cath lab. Initial experience with this protocol has found it saves about 10–20 minutes. Such a time saving initially appears modest. In fact, it is at least as much time has been saved by other prehospital and hospital practices—associated with time savings of 8–19 minutes—that have been judged to be significant contributors to efforts to meet a 90 minute door-to-balloon deadline [91]. There is growing recognition of importance of transporting ST-elevation myocardial infarction (STEMI) patients for primary percutaneous intervention (PCI). For example, a consortium US panel of US EMS medical directors has recently been identified as an evidence-based benchmark for quality prehospital care, the transport of STEMI patients to primary PCI within 90 minutes of EKG diagnosis [92]. Recent meta-analysis confirms the substantial outcomes benefits, in terms of both mortality and morbidity (including from stroke), of timely transfer of STEMI patients for mechanical reperfusion [93]. It is clear that, for some regions and their patients, HEMS provides a vital capability to meet this benchmark.

The authors of a logistics study from the University of Wisconsin [22] noted that HEMS and fast transport are occasionally critical even for patients who are not profoundly unstable, but who may need time-windowed cardiac or stroke therapy. In assessing average transport times from their 20-hospital network, the investigators found that for all hospitals, the average HEMS total transport time over the study period was at least as good as the best ground transport time, and this took into account the fact that for many hospitals ground EMS was on site at the time of transport. Furthermore, the authors found that there were clinically significant time savings for all institutions: patients at close-by hospitals accrued an average of 10 minutes' time savings, while those from further-out hospitals had HEMS transport times of up to 45 minutes shorter than achievable by ground transport.

### 3.7. Minimization of Out-of-Hospital Time

As an additional facet to the time issue, the issue of “out-of-hospital” time (for interfacility transports) should be considered separately from the general issue of “pretrauma center time.” Even if a HEMS service takes longer than local ground units to respond to a community hospital patient requiring transport to a tertiary care center, in most cases the actual time spent in patient transport is much less for HEMS patients. In one study, for instance, even though the overall time characteristics of HEMS were not significantly better than ground EMS performance, the actual out-of-hospital time saved by HEMS use averaged 20 minutes (58 minutes for HEMS versus 78 minutes for ground transport) [67]. In some patients—especially those who are in tenuous condition or who may require difficult interventions in the event of deterioration—the minimization of time spent in the relatively uncontrolled out-of-hospital transport environment is an admirable goal. As an example, in some areas high-risk obstetric patients are often transported by air (helicopter or fixed wing) to minimize out-of-hospital times (and decrease the chances of intratransport delivery). In Japan, for instance, reduction in out-of-hospital times averaged over 100 minutes for high-risk obstetric patients transported by air as compared to ground; the reduction in out-of-hospital times was theorized by the authors to contribute to good maternal and fetal outcomes in their transported population [94].

### 3.8. Direct Transport to Specialized Centers (for Primary/Scene Missions)

As considered from the point of view of the patient, the benefit of direct transport to specialized centers relates to the debate about whether community or tertiary care hospitals provide better care. For some diagnoses, such as trauma or acute coronary syndromes, a strong argument can be made for bypassing community hospitals in favor of direct transport to larger, higher-volume centers (and perhaps more capabilities such as primary percutaneous coronary intervention) [95]. For areas in which there is no trauma center, air medical scene response for direct transport to the trauma center is often the best course [31]. In fact, there is strong evidence basis to suggest that, for blunt trauma patients, bypassing community hospitals (including HEMS-executed bypass) in favor of direct transport to Level I trauma centers has a significant and positive impact on outcome [70, 96–102]. Experts with no interest in the HEMS debate have noted that “it is beneficial for a patient to be taken to a designated trauma center rather than a nontrauma community hospital” [99]. More recent evidence finding a clear correlation between trauma center status (Level I or Level II) and adherence to well-accepted Traumatic Brain Injury Guidelines concludes that direct transport for brain-injured patients to trauma centers will improve outcome [103]. There are also substantial implications for HEMS in the (controversial) studies that reveal a distinctly improved outcome from transport to Level I (as compared with Level II) trauma centers [70].

The most notable recent paper in the HEMS literature, a nationwide study of over 250,000 adult and pediatric scene trauma transports from the National Trauma Data Bank (NTDB), is also tied to the Level I versus other-level trauma centers [49]. The paper's finding that HEMS was associated with a 22% improvement in mortality was discussed by the authors as possibly indicating that HEMS afforded access to Level I (and to a lesser extent, Level II) trauma center care. A few months after the publication of the NTDB study by Brown et al., the results were confirmed in a study of the same NTDB 2007 dataset, from the Centers for Disease Control; the fact that the CDC study focused only on adults probably explains its larger point estimate (39%) for HEMS-associated mortality benefit [104]. The very large-scale aspect of the NTDB studies that make results so compelling renders it impossible to tease out specifics—but even if HEMS transport was nothing more than a marker for capability to get patients to high-level trauma care, the results remain compelling in the “real world.”

As a general rule, use of HEMS for direct transport to tertiary care is commonly used for trauma patients and less commonly used for other patient categories. Data from the Centers for Disease Control and elsewhere has confirmed that, for the general population of injured patients, trauma center care (i.e., appropriate triage) results in substantially reduced mortality [69, 105]. In a study focusing on the subset of patients with severe traumatic brain injury, and with methodology adjusting for hypotension, age, GCS, and pupillary reactivity, a group of investigators from New York State found that direct transport to a trauma center provided a clear outcomes benefit [106]. Authors of that study point out that the Guidelines for Prehospital Management of Traumatic Brain Injury call for direct transport to high-level care, when severe brain injury is present (GCS < 9) [107]. It is also well known that delays at nontrauma centers, which can result from a variety of factors such as specialist nonavailability, prolong pretrauma center times and worsen injured patients' outcomes [108–111]. Such reports may be reasonably expected to increase utilization of HEMS for such “direct” transfers from scenes to trauma center care. Trauma triage and systems experts have found that patients with head injuries, and those patients with physiologic findings meeting trauma triage criteria, had significantly better outcome when treated at regional centers as compared to area (Level 2) trauma centers or nontrauma centers [112, 113]. For adult and pediatric trauma patients who are initially treated at nontrauma centers, transfer to Level I centers is associated with substantial improvement in outcome (mortality odds ratio 0.62 as compared to patients kept at nontrauma centers); thus interfacility transfer (which will occasionally be via HEMS) is warranted and appropriate [102]. Henry et al. write “the considerable improvement in survival raises the question of whether patients meeting these physiologic criteria with improved outcomes should be transported directly to regional centers, even if that means bypassing an area trauma center” [112]. 

On the nontrauma front, suggestion of potentially growing indications for HEMS “scene” transports of noninjured patients is provided by an evolving literature consisting of both case series (e.g., for primary percutaneous intervention) and sporadic reports (e.g., scene transport to neurological centers for lytic therapy for ischemic stroke) [27, 47, 114]. A Japanese report finds that, compared to ground ambulance transport, HEMS use in their particular system is associated with a half-hour's decrement in times to angiographic evaluation and intervention [114]. A recent preliminary report on simultaneous HEMS dispatch and tertiary care hospital cardiac cath lab activation, by ground prehospital providers diagnosing STEMI during transport to a community hospital, found that the time at the referring hospital was reduced from 79 to 31 minutes [115]. The economic factors driving the growing trend towards regionalization of many critical care services will continue to spur investigation into routine use of HEMS for indications that would be considered novel in past years. Early indications that outcomes are improved with stroke care in specialized centers may add to the efforts to integrate transport plans into regional care for this disease [116]. 

While the integral nature of HEMS as part of a system may make it difficult to delineate the specific outcomes contribution made by the helicopter, the HEMS effect is no less important. When considering a report [117] that HEMS integration into a cardiac care system allows for diagnostic catheterization to be performed at community hospitals, with rapid air transport for interventional procedures when needed, it is not easy to either prove or refute the critical nature of HEMS for patient outcomes. Similarly, it is not easy to discount the potential benefit to stroke patients, when reviewing a study from north Florida demonstrating the effective integration of HEMS into the stroke system, with resultant extension of the “reach” of advanced stroke care such as thrombolytic therapy [87]. The same logic holds true for injured patients undergoing air transport to Level I centers [118]. In these cases, direct transport to specialized centers likely benefits many patients. Additionally, the judicious integration of HEMS into a system of care has high probability of accruing benefits to the region itself. These benefits are among other “systemwide” benefits of HEMS, and are considered in the next section.

## 4. Possible HEMS Benefits to Systems

It goes without saying that if HEMS is associated with mortality (or significant morbidity) improvement, then it benefits a regional EMS system to have access to such a service. Whether the EMS region is dealing with increased interfacility transports as a result of implementation of “inclusive” trauma systems [100] or more frequent HEMS use for stroke patients, air medical transport clearly has a vital role in regionalization of care. While patient-centered thinking should be paramount, some logistic and economic considerations represent very important potential utilities for HEMS services.

### 4.1. Extension of Advanced Level of Care Throughout a Region

Some of the above-mentioned benefits to patients also apply as advantages to regions and EMS systems. For example, HEMS may allow an EMS system ability to provide for early ALS in isolated and/or difficult-to-reach areas which otherwise would be poorly covered. In pointing out that HEMS can cover roughly the geographic area of seven ground ALS ambulances, Hankins [53] has written that “this kind of coverage, in many areas of the country, provides advanced care where it is not otherwise available.” Others, considering the US trauma system as a whole, have agreed that at least in some areas of the US, the extension of trauma regional care provided by HEMS is critical [49]. Analysis of the economics of covering a widespread region using a small number of aircraft, as compared to a large number of ground vehicles dispersed in such fashion as to assure equivalent response times, is complex; preliminary analysis has suggested that HEMS is actually no more expensive than the multiple-ground-unit alternative.

In fact, limitation of the HEMS versus “highly trained ground EMS” argument to economic considerations ignores the fact that EMS cannot simply fiat into the ground personnel “high level of training” that comes with concentrated training and experience accorded to HEMS crews. Recent literature suggests that even with major emphasis on training, some ground EMS systems have had efficacy difficulties when neuromuscular blockade-assisted ETI protocols were instituted. In at least two regions, neuromuscular blockade-assisted intubation by ground EMS was sufficiently problematic that the practice was discontinued. Contrasting this with the 95–98% intubation success rates regularly reported by HEMS services, HEMS proponents make sound arguments that HEMS is a reasonable means for a given EMS region to provide a high level of care to a large area.

HEMS may offer benefits even to patients already at (smaller) hospitals [33]. This is most likely true in rural settings in which local facilities may be staffed by individuals with relatively little experience with trauma or other critical illnesses [32]. In trauma, for instance, the lack of ready availability of surgical subspecialists (e.g., neurosurgeons) is translating to an increasing inability of non-Level I centers to care for injured patients [108]. Trauma triage experts have labeled as “undertriage” any instance of transporting to any hospital lacking emergency access to neurosurgeons, a traumatic brain injury patient with potential for requiring neurosurgical monitoring or craniotomy [108]. 

The issue of regionalization of trauma care is well known to acute care physicians, but recent data have clarified the importance of capabilities to get injured patients to a trauma center. In fact, a 2008 consortium panel of US metropolitan EMS medical directors emphasized the importance of transporting patients for trauma center care if they have ISS > 15 (number needed to treat to save one life: 11) [92]. As trauma systems mature, there is obviously a role for HEMS in the occasional transport of patients to insure that life-saving care is available to more patients throughout a region.

### 4.2. Provision of ALS “Backup” for Parts of an EMS System Which Have Limited ALS Coverage

In addition to providing ALS-level care to geographically remote areas, HEMS can offer a means for relatively isolated areas to get patients to tertiary care centers without necessitating removal of scarce ground ALS resources from the region. At least one paper [119] has specifically identified that one major reason rural areas use HEMS is that they perceive they are unable to cope with losing their limited ground ALS coverage for what can be a 5-hour round trip. For better or for worse—use of HEMS for patients with noncritical illness or injury may not be in the best interest of the system as a whole—some regions have come to rely on HEMS as a means to assure that they will not lose ALS coverage for hours, every time a patient requires ALS-level transport to a distant receiving hospital. As an added benefit, the use of helicopters for longer-distance transports of critical patients can reduce the risks associated with prolonged red-lights-and-siren ground EMS transports [53].

### 4.3. Minimization of Transport Times

The utilization of HEMS for some transports, and its resultant streamlining of out-of-hospital times, can benefit EMS systems as well as individual patients. Examples of such benefits include faster turnaround and greater availability for transport. The overall transport time minimization discussed earlier, with respect to trauma, cardiac, and stroke care, should also be viewed as a system benefit. It should be kept in mind that the total time savings accrued by HEMS are not just beneficial for scene flights; interfacility patients also get to definitive care more quickly with HEMS [22, 72]. 

### 4.4. Direct Transport to Specialized Centers

Like some of the other advantages potentially accrued by individual patients, this benefit can also be said to be accrued by an EMS system. One purpose of the EMS regional authority is to provide the optimal prehospital and out-of-hospital transport setup; so patients can get to where they need to be. In many cases, this will be the closest facility; in such circumstances ground transport will usually be a preferable alternative. However, some patient populations have definite, probable, or possible indications for direct transfer to a specialized center with bypassing of community facilities. Despite the ongoing debate with respect to “inclusive” versus “exclusive” trauma systems—a debate which entails points outside the scope of this discussion—the fact remains that care at Level I centers improves morbidity and mortality outcomes for many patient types [69, 73, 120–122]. Furthermore, HEMS studies commonly identify significant mortality benefit from direct transportation from scenes to tertiary care (rather than initial ground transport to a “stabilizing” hospital first) [101]. Emerging literature makes compelling arguments, from perspectives of both outcomes and cost (e.g., preventing dual workups), for direct transport of pediatric trauma patients to specialized centers [123].

 As regionalization of care continues to evolve, EMS systems will doubtless play a major role in both primary (i.e., scene) and secondary (i.e., interfacility) transport of an increasing number of patients requiring specialized care. In fact, a 2010 study revealed that centralization of cardiac catheterization resources, with appropriate buildup of EMS transfer systems, is significantly more cost effective than construction of multiple cardiac catheterization centers; the same authors note that 20% of Americans live more than an hour away (by ground) from a cardiac catheterization center [124]. HEMS' role in such regionalization is not yet fully characterized, but the existing literature renders clear the fact that air medical transport does have some role in optimizing regionalization. 

### 4.5. Transport Flexibility in Overloaded Hospital Systems

The helicopter offers advantages of being flexible with respect to receiving center; not much time is lost in changing the receiving hospital destination if it is close by, and the helicopter's speed and “legs” can often bring relatively distant hospitals into play if local facilities are overloaded. Though the obvious benefit to this (for EMS systems) relates to unusual circumstances such as disasters [125, 126], the current environment of hospital and E.D. overcrowding renders the receiving hospital flexibility of HEMS a potentially useful thing.

With the advent of increasing problems due to ambulance diversion, the transport flexibility provided by HEMS has additional advantage. Since ambulance diversion problems can often result in a given ground EMS unit being out of service for an extended period (i.e., while it is performing a longer-distance transport) [99], the aircraft may be able to “back up” the ambulance by either performing the transport or being available while ground EMS is out of service. With increasing evidence demonstrating trauma mortality rates increasing when trauma centers' EDs are on diversion [127], the HEMS unit can serve as a life-saving method for “decompressing” the overtaxed ED. In fact, the utility of HEMS to distribute the patient load, already noted for its potential value in disaster and mass casualty incidents, may be applicable in some areas' Level I trauma centers on an increasingly frequent basis [125]. The loss of availability of rotor-wing transport has been recognized as a potential mediator of increased mortality due to decreased capability to execute interfacility transports [73].

### 4.6. Ability to Perform Unusual and Ad Hoc Activities

While noone questions the flexibility and capabilities of ground EMS units, the nature of the helicopter lends itself to utility in unusual circumstances. For example, in the unusual case where a medical expert or team needs to be transported to the patient, the speed and logistical capabilities of the helicopter may be useful [128]. The utility of HEMS in disaster and mass casualty incidents is well described [125, 129]. In fact, during the London subway bombings of 2005, the London HEMS aircraft flew at least 25 missions—none of them patient transports, but rather transportation of medical care teams to incident sites. Given the traffic situation in London at that time, the HEMS was judged to be a vital part of the emergency response (personal communication, Dr. David Baker of the UK's Health Protection Agency, 21 June 2007).

Additional reports from around the world outline unusual use of HEMS resources, which do not justify expense for an aircraft, but which nonetheless represent (in conglomeration) a potentially significant illustration of HEMS' ad hoc utility. For example, the French have reported HEMS response to cruise ships at sea, enabling time critical and successful lytic therapy for stroke [130]. 

In addition to transporting people, helicopters have been occasionally used to rapidly transport vital supplies or equipment (e.g., prostaglandins to a neonate with a ductus-dependent lesion). Another “unusual” activity that may for some regions be appropriate for HEMS is performance of research in the out-of-hospital setting. Particularly in rural regions, where the HEMS crews arrive at patients (both at scenes and at referring hospitals) long before the patient will get to Level I care, it has been suggested that a small cadre of air medical personnel can be trained to intervene/enroll patients in clinical studies with a narrow time window [74, 131].

## 5. Conclusion

This paper has attempted to overview the important questions of HEMS' possible benefits to patients and to healthcare systems. The potential benefits of HEMS must be considered by policymakers and others providing HEMS use guidance, with other parameters not discussed in this paper (e.g., triage, utilization review, cost effectiveness, and safety).
